# Association mapping through heuristic evolutionary history reconstruction-application to GAW15 Problem 3

**DOI:** 10.1186/1753-6561-1-s1-s131

**Published:** 2007-12-18

**Authors:** Alexander Platt

**Affiliations:** 1 Department of Molecular and Computational Biology, University of Southern California, 1050 Childs Way, Los Angeles, California 90089-2910, USA

## Abstract

This paper presents a novel method of identifying phenotypically important regions of the genome. It involves a form of association mapping that works by summarizing properties of the ancestral recombination graph (ARG) of a sample of unrelated phenotyped and genotyped individuals. By breaking the sample into many small sub-samples and averaging the results, it becomes computationally tractable to measure the degree to which the evolutionary history of any locus is consistent with the distribution of the phenotypes in the sample. Analysis of simulated rheumatoid arthritis data demonstrates the efficiency and effectiveness of this method in identifying loci of large phenotypic effect.

## Background

Explicit reconstruction of the evolutionary history of a sample can provide a powerful tool for detecting genetic contributions to phenotypic variation in a population. This new method exploits the fact that an allele shared among individuals in a sample that contributes to similarities of those individuals' phenotypes must have originated in an ancestral lineage shared only by those individuals. Therefore, regions of the genome containing genes that contribute to the differences among individuals in the sample will tend to have evolutionary histories that reflect the phenotypic distributions of those individuals. Other regions not contributing to the phenotype in question, will, due to recombination, have histories that are uncorrelated with the distribution of phenotypes.

Cladistic analyses in association studies are a concept that date back to the late 1980s [1], and have been implemented several times since then [2-7]. Due to the complexities of within-population evolutionary histories, they have tended to employ clustering algorithms which result in decidedly non-evolutionary cladograms of individuals.

This paper considers the case of phased haplotype data drawn from a population of binary phenotypes. Given infinite computing power, the process would involve using the genetic data to infer the entire ancestral recombination graph (ARG) of the sample. This would give the entire set of evolutionary relationships of every piece of every haplotype with every other piece of every haplotype. From there it would be a simple matter to pick the branches of the graph that most consistently divide the sample into groups of cases and controls, and then to infer which segments of the haplotypes those branches describe. This is not a feasible approach with current or developing technology, so it is necessary to describe a heuristic method for capturing the most important signal from this graph without reconstructing it in its entirety.

What makes ARGs so difficult to reconstruct is that the number of possible ARGs for a sample increases exponentially with the number of haplotypes in the sample. While it is not difficult to infer an optimal ARG for a sample of extremely small size, it quickly becomes intractable for sample sizes even in the tens of haplotypes. This method capitalizes on the relative ease of estimating ARGs for small sample sizes by successively computing ARGs for many small sub-samples from within the set of haplotypes. For each site in each sub-sample, it is easy to determine whether that ARG correctly divides the sub-sample cases from the sub-sample controls. The frequency across sub-samples is then the test statistic for determining the general trend in the topology of the overall ARG at each site. This statistic is a measure of the degree to which the history of a locus is consistent with the resulting phenotypic distribution of the haplotypes and will be referred to as the consistency of the sample.

## Methods

### Algorithm

This algorithm is an iterative process of selecting and analyzing subsets of the data. Each iteration starts with randomly selecting quartets of haplotypes composed of two control haplotypes and two unrelated case haplotypes. The program *beagle *[8] is used to infer the minimum number and location of recombinations necessary for each quartet to conform to an infinite sites model of evolution. *Beagle *scans the haplotypes for pairs of markers at which all four possible gametes are present. Without invoking recurrent mutation, this pattern indicates the presence of a recombination event somewhere between such marker pairs. *Beagle *assigns a minimum set of locations of recombination events that satisfies all such pairs. These are used to divide the haplotypes in the quartet into a set of genomic segments each consistent with a single tree for an evolutionary history.

**Figure 1:**
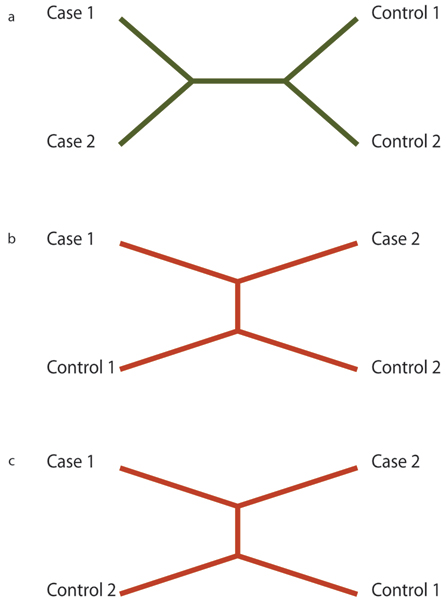
**Possible tree topologies**. Figure 1a represents a history consistent with the phenotypic distribution. Figures 1b and 1c do not.

Next, each of these segments is passed to the *pars *program in the package PHYLIP [9] to generate a single most parsimonious tree reflecting the history of that segment. This is the inferred evolutionary tree that minimizes the number of mutation events to describe all of the differences between the sequences. Of the three possible topologies for an unrooted tree with four taxa and no ambiguities (Fig. 1), only one will contain a branch that separates the two cases from the two controls. Segments generating a tree with this topology are considered "consistent". Either of the other two topologies is considered "inconsistent". Segments that do not unambiguously generate a maximum parsimony tree that is either consistent or inconsistent are thrown out.

After each segment is analyzed, the frequency across quartets in which each site finds itself in a consistent segment is calculated. This frequency, the consistency of the sample, is used as the test statistic. Elevated levels of consistency correspond to sites better able to explain the differences between case and control phenotypes. The amount of information learned from the data increases with each iteration but does so with asymptotically diminishing returns once every possible quartet has been picked.

### Assessing significance

The significance of results is addressed through classical hypothesis testing. The data are modeled as being drawn at random from two populations, one of case haplotypes and one of control haplotypes. Every mutation in the history of the sample distinguishes between two alleles and creates two types of haplotypes, one with the ancestral allele at that site and one with the derived allele. If a particular mutation contributes to the probability of disease, then haplotypes containing that allele will be over-represented in the case population. The null hypothesis is that the two types are represented with equal frequency in both the case and control populations.

A given data set can be summarized as having *K*_1 _case haplotypes with *a*_1 _copies of one type of haplotype and (*K*_1 _- *a*_1_) of the other, and *K*_2 _control haplotypes with *a*_2 _and (*K*_2 _- *a*_2_) copies of the two types of haplotypes respectively. As all the trees are unrooted, it is not necessary to identify which type is ancestral and which is derived. Therefore, which type is labeled (*a*) is an arbitrary designation.

The probability of a randomly drawn quartet being counted as consistent, *p*(*a*_1_, *a*_2_, *K*_1_, *K*_2_), can be calculated as

$$\begin{array}{l} \frac{a_{1}^{2}-a_{1}}{K_{1}^{2}-K_{1}}\frac{\left(K_{2}-a_{2})(K_{2}-a_{2}-1\right)}{K_{2}^{2}-K_{2}}+\frac{a_{2}^{2}-a_{2}}{K_{2}^{2}-K_{2}}\frac{\left(K_{1}-a_{1})(K_{1}-a_{1}-1\right)}{K_{1}^{2}-K_{1}}\\ +\frac{1}{3}\left[\frac{a_{1}^{2}-a_{1}}{K_{1}^{2}-K_{1}}\left(1-\frac{\left(K_{2}-a_{2})(K_{2}-a_{2}-1\right)}{K_{2}^{2}-K_{2}}\right)+\frac{\left(K_{1}-a_{1})(K_{1}-a_{1}-1\right)}{K_{1}^{2}-K_{1}}\left(1-\frac{a_{2}^{2}-a_{2}}{K_{2}^{2}-K_{2}}\right)\right]\\ +\frac{1}{3}\left[\frac{\left(K_{2}-a_{2})(K_{2}-a_{2}-1\right)}{K_{2}^{2}-K_{2}}\frac{2(K_{1}a_{1}-a_{1}^{2})}{K_{1}^{2}-K_{1}}+\frac{\left(K_{1}-a_{1})(K_{1}-a_{1}-1\right)}{K_{1}^{2}-K_{1}}\frac{2(K_{2}a_{2}-a_{2}^{2})}{K_{2}^{2}-K_{2}}\right]. \end{array}$$

This is the probability of selecting a quartet composed of two haplotypes of one type from the control set and two haplotypes of the other type from the case set (which are the quartets that contribute to the signal in the data) plus 1/3 times the probability of selecting a quartet that contains three or four haplotypes of the same type and one or zero of the other (these quartets are uninformative and contribute only random noise).

The probability of a site being counted as consistent *x *times after *N *quartets have been analyzed should be calculated as

$$\int_{0}^{1}\sum_{a_{1}=0}^{K_{1}}\sum_{a_{2}=0}^{K_{2}}\left(f(\alpha )g\left(a_{1},K_{1},\alpha \right)g\left(a_{2},K_{2},\alpha \right)\left(\begin{array}{c} N\\ x \end{array}\right){\left(p(a_{1},a_{2},K_{1},K_{2})\right)}^{x}{\left(1-p(a_{1},a_{2},K_{1},K_{2})\right)}^{N-x}\right)d\alpha .$$

This is essentially a binomial sampling probability with *p*(*a*_1_, *a*_2_, *K*_1_, *K*_2_) being the chance of success on a particular trial, summed over all the possible values of the random variables *a*_1 _and *a*_2 _times their probability densities, *g*(*a*_1_, *K*_1_, *α*) and *g*(*a*_2_, *K*_2_, *α*), which are the binomial sampling probabilities of drawing *a *of the arbitrarily labeled haplotypes in *K *samples from a total population where the labeled type of haplotype is at frequency *α*.

Because the probability densities of *a*_1 _and *a*_2_ depend on the random variable *α*, the whole statement has to be integrated over all possible values of *α *times its probability density, *f*(*α*). Unfortunately, *f*(*α*) is unknown, so exact marginal probabilities cannot be calculated directly. However, for values of consistency above 1/3, which are the only ones of interest, the likelihood of the null hypothesis is at a maximum when *α *= 0.5, so assuming *α *= 0.5 and calculating *p*-values by summing the probabilities of finding the observed or higher number of consistent quartets is conservative.

## Results

**Figure 2:**
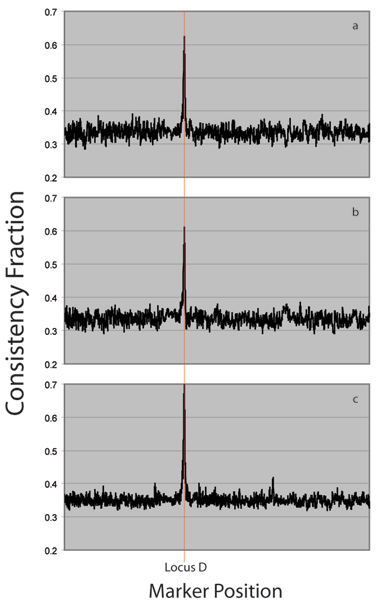
**Consistency analyses on simulated data**. Figure 2a shows consistency measures after 1000 iterations on a full simulated chromosome. Figure 2b uses 10% of the data. Figure 2c uses 1% of the data.

Applied to the high-density simulated SNPs of Replicate 1 of the Genome Analysis Workshop 15 Problem 3 data set, the algorithm correctly identifies Locus D as a site of great importance in determining simulated disease status. Figure 2 shows calculated consistency values along the chromosome after analyzing 1000 quartets. Using the entire data set of 3000 case haplotypes and 2000 control haplotypes gives an estimated *p*-value of less than 10^-308 ^at the correct locus. This is the lowest *p*-value for the chromosome, and in fact, the *p*-value may be considerably lower than this because smaller values are difficult to compute accurately. The actual numerical value has little meaning beyond demonstrating the vanishingly small probability of getting such a signal by chance. Figures 2b and 2c represent the same analysis carried out on smaller random subsets of the data. In Figure 2b only a random subset of 10% of the haplotypes are used. The algorithm still correctly identifies the simulated locus and estimates a *p*-value less than 10^-65^. In comparison, a simple chi-squared test yields a *p*-value of 10^-52 ^at the correct locus. Figure 2c uses a random subset of 1% of the haplotypes, still correctly identifies the proper locus, and gives a *p*-value of less than 10^-11^. The chi-squared test on these data gives a *p*-value of 10^-5^.

## Discussion

There are several practical matters to consider in applying this method. The first is that while the simulated data analyzed in this paper were given as fully phased haplotypes, most real data are only known as unphased genotypes. Therefore, an additional step of estimating phase is necessary. There are several widely used algorithms for accomplishing this, such as fastPHASE [10] or HAPLOTYPER [11]. These algorithms use the population genotype frequencies in the samples to estimate the haplotype frequencies contributing to the sample. They are generally very accurate over short ranges but can introduce errors, essentially artificial recombination events between haplotypes, between markers with very little linkage disequilibrium. These types of errors do not influence consistency estimates much at all, because all of the information used in estimating consistency comes from closely linked markers. Furthermore, introducing artificial recombinations into the data set can only increase the type II error rate, not the type I error rate.

Similarly, this method is conservative to errors introduced by violations of the infinite sites model of evolution. *Beagle *invokes the infinite sites model to infer the existence of a recombination event. Recurrent mutation could cause *beagle *to include extra recombination points. The only detriment to this is that *pars *must now estimate two trees from the information that it would otherwise use to estimate just a single tree. This would cause a very local reduction in power but cannot create false positives. A massive departure from the infinite sites model could mean that the trees produced by *pars *are poor reconstructions of the true historical events. However, this too should only reduce the sensitivity of the test, not inflate the type I error rate.

The consistency estimate is unbiased for all values of *N*. As *N *increases, the *p*-value of any consistency estimate above 1/3 decreases. There are diminishing returns as *N *becomes a sizeable fraction of all the possible quartets. As the number of possible quartets is generally far larger than is reasonable (or worthwhile) to examine, a practical guideline would be to continue running the algorithm until effects of a minimally interesting consistency value are significant.

## Conclusion

Population genetic data arise through a well understood and well modeled process of evolution. In association mapping studies, treating each locus and each haplotype as independent, unrelated trials, as in the chi-squared test and other simple regression analyses, robs an investigator of much useful information in her or his data and opens the door to spurious results. Making use of our understanding of the process through which these data are created allows us to fashion more powerful and more reliable methods. The method of consistency shows that even a simple algorithm using piecewise approximations of evolutionary histories can offer a great increase in statistical power. Additionally, statistics built on biological models can represent easily intuited biological properties. Unlike a chi-squared statistic, consistency is itself a measurement that relates directly to the biological phenomenon that it tests.

## List of Abbreviations

ARG: ancestral recombination graph

## Competing interests

The author(s) declare that they have no competing interests.
